# Involvement of the central nervous system in acute lymphoblastic leukemia: opinions on molecular mechanisms and clinical implications based on recent data

**DOI:** 10.1007/s10555-020-09848-z

**Published:** 2020-01-22

**Authors:** Lennart Lenk, Ameera Alsadeq, Denis M. Schewe

**Affiliations:** 1 Department of Pediatrics I, ALL-BFM Study Group, Christian-Albrechts University Kiel and University Medical Center Schleswig-Holstein, Kiel, Germany; 2 Institute of Immunology, Ulm University Medical Center, Ulm, Germany

**Keywords:** Central nervous system, Acute lymphoblastic leukemia, CNS involvement, Blood-brain-barrier, Tumor dormancy, Immunotherapy

## Abstract

Acute lymphoblastic leukemia (ALL) is the most common childhood cancer. One of the major clinical challenges is adequate diagnosis and treatment of central nervous system (CNS) involvement in this disease. Intriguingly, there is little solid evidence on the mechanisms sustaining CNS disease in ALL. Here, we present and discuss recent data on this topic, which are mainly derived from preclinical model systems. We thereby highlight sites and routes of leukemic CNS infiltration, cellular features promoting infiltration and survival of leukemic cells in a presumably hostile niche, and dormancy as a potential mechanism of survival and relapse in CNS leukemia. We also focus on the impact of ALL cytogenetic subtypes on features associated with a particular CNS tropism. Finally, we speculate on new perspectives in the treatment of ALL in the CNS, including ideas on the impact of novel immunotherapies.

## CNS involvement in ALL: a clinical challenge

Acute lymphoblastic leukemia (ALL) is the most frequent cancer in children worldwide. National and international collaboration brought forward chemotherapy-based treatment protocols yielding ~ 80–90% cure rates [1]. However, these encouraging results come at the cost of therapy toxicity. Furthermore, one in five children will relapse during the course of the disease, which is associated with an inferior prognosis [1]. One of the major clinical challenges is adequate diagnosis and treatment of central nervous system (CNS) involvement, which is detected in about 3–5% of patients at initial diagnosis and 30–40% of patients at relapse [2, 3]. The gold standard to assess CNS involvement is the detection of leukemic cells in the cerebrospinal fluid (CSF) after lumbar puncture. However, the observation that most CNS relapses occur in patients initially diagnosed as CNS-negative displays the urgent need for more precise diagnostic approaches [4]. Novel strategies aiming to improve sensitivity of CSF diagnosis *via* qPCR or flow cytometry analyses have not yet entered clinical routine, mainly due to methodological challenges and the lack of validation [5–7]. Early autopsy studies suggest that the majority of leukemia patients would develop CNS disease during the course of the disease [8]. Hence, irrespective of the initial CNS status, all patients are treated with potent intrathecal prophylactic chemotherapy (methotrexate in most protocols), for which an association with neuronal injury and leukoencephalopathy has been shown [9]. Currently known risk factors for CNS involvement in ALL include peripheral hyperleukocytosis upon diagnosis and a T cell immunophenotype [3]. In B cell precursor (BCP)-ALL, certain cytogenetics like the t(1;19) translocation leading to the *E2A-PBX1* fusion gene and the t(9;22) translocation causing the *BCR-ABL1* fusion are associated with a higher incidence of CNS leukemia [10–12]. Furthermore, a mixed lineage leukemia (*MLL* or *KMT2A*)-rearranged cytogenetic background is also a risk factor for CNS disease [13]. Patients at high risk for CNS infiltration receive additional cranial irradiation in some protocols, which further increases the risk for neurocognitive deficits and secondary malignancies [2]. Hence, besides conclusive diagnostic markers indicating CNS involvement novel target molecules for specific eradication of ALL cells in the CNS have to be established.

The specific mechanisms and molecules fostering CNS disease in ALL are widely unknown. Here, we will highlight the mechanisms and molecular background of CNS disease in ALL based on recent findings. We will also present some opinions on potential novel clinical applications in the future.

## Accessing the CNS: sites and routes of leukemic infiltration

The question of how leukemia cells infiltrate the CNS was initially raised in the early seventies [8]. Due to the limitations of current model systems to depict the complexity of the CNS, the investigation of the means by which leukemic cells infiltrate the CNS remains a difficult endeavor. Nevertheless, the recent years have brought forward some interesting approaches that shape a more detailed image of the anatomic entry routes exploited by leukemic cells in order to invade the CNS.

### Anatomical structures with potential involvement in leukemic infiltration

**Fig. 1:**
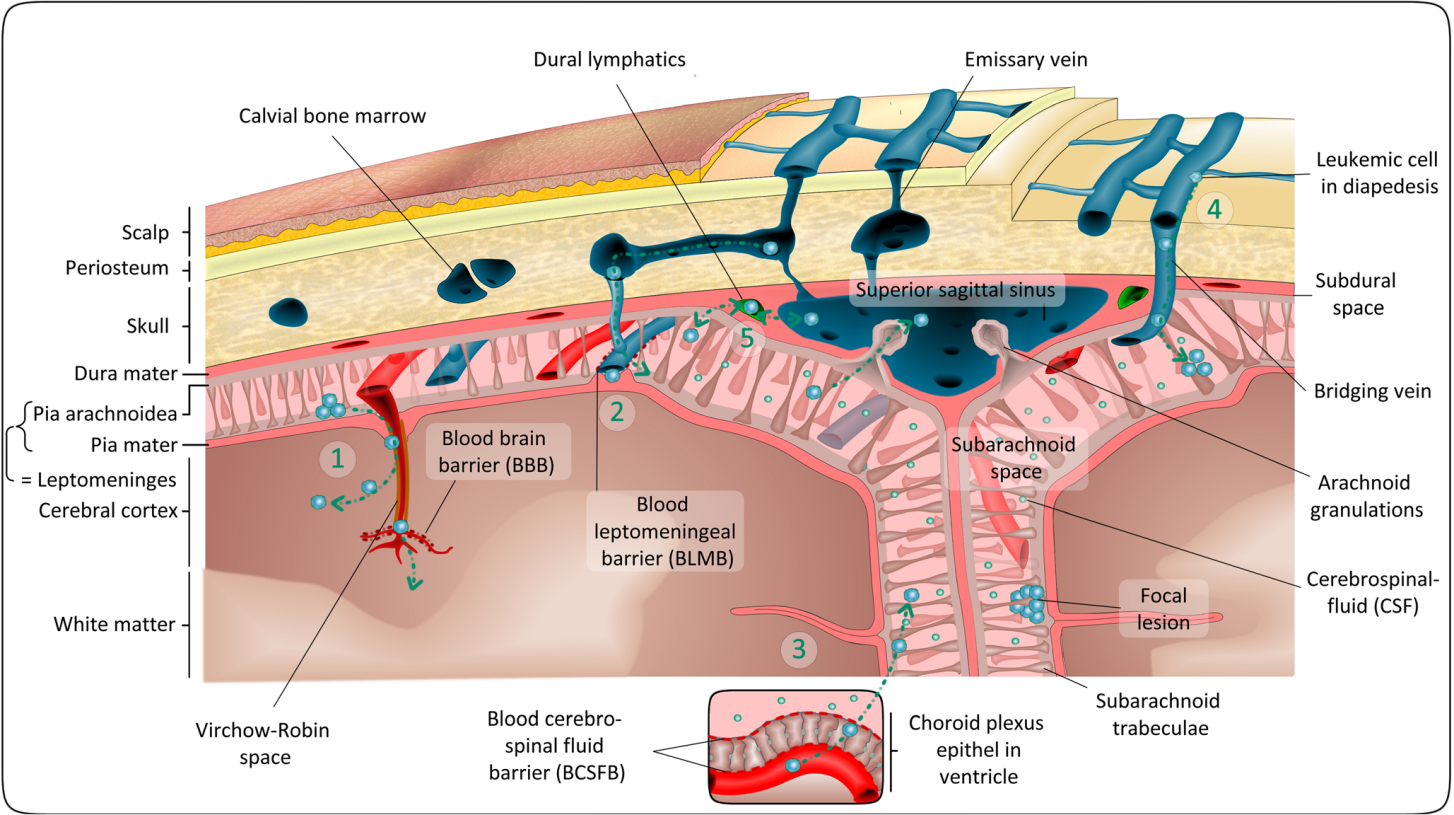
Potential entry routes and sanctuaries for ALL cells in the CNS. The major locations of CNS involvement in ALL are the meninges consisting of the dura, arachnoid, and pia mater. In the subarachnoid space between arachnoid and pia, ALL cells may either spread or persist as focal lesions in which they may not be detected *via* lumbar puncture. Entry into the meninges and the subarachnoid space can occur *via* different routes. Entry from the vasculature into the CNS may occur *via* the blood brain barrier (BBB) of microvessels in the brain parenchyma (1), the blood leptomeningeal barrier (BLMB) on the surface of the pia mater (2), or the blood cerebrospinal fluid barrier (BCSFB) (3). The BCSFB is situated in the choroid plexus epithelium, which also produces the cerebrospinal fluid (CSF) in the ventricles of the brain. A recent report suggests that ALL cells may avoid these barriers and directly travel into the subarachnoid space along the surface of bridging veins traversing the skull and meninges (4). Moreover, dural lymphatics draining leukocytes out the parenchyma and subarachnoid space may represent an additional route for leukemia cells to enter and leave the subarachnoid space (5)

The CNS comprises the brain and the spinal cord (Fig. 1). Protection of this highly sensitive organ is provided by a three-layered structure collectively termed the meninges, which moreover support the vessels and embed the CSF transport cavities. The meninges consist of the outer dura mater, which is separated from the inner meningeal layers by the subdural space. The inner meningeal layer comprises the arachnoid mater and the pia mater, which are together referred to as the leptomeninges and coat the brain parenchyma. The subarachnoid space, a space between arachnoid and pia, harbors the cerebral vessels and the CSF (outer liquor space), and therefore also represents the space which is accessed during diagnostic lumbar puncture. Furthermore, the arachnoid mater exhibits protrusions called pacchionian granulations (arachnoid granulations) that reach into the dural venous sinuses and resorb CSF from the subarachnoid space into the blood circulation. The CSF contributes to mechanical cushioning and metabolic homeostasis of the CNS. It is produced by the choroid plexus, an epithelial layer located within the walls of the ventricles (inner liquor space). Outer and inner liquor spaces are in contact in the region of the fourth ventricle. The high metabolic requirements of the CNS are met by a complex vascular system. Blood supply of the CNS occurs via the vertebral arteries and the internal carotid arteries. The arterial blood supply of the brain and the meninges are separated, whereas the drainage of venous blood of both systems happens via the cranial sinuses. Moreover, the meninges are traversed by bridging veins. These vessels pass through the vertebral and calvarial bone marrow and the subarachnoid space, and therefore represent a direct connection between the superficial veins of the skull and the meningeal system.

Interfaces between vessels and CNS structures represent a complex barrier system that in physiological conditions accounts for the selective and controlled flux of molecules and cells into the CNS. In the context of leukemic CNS infiltration, the endothelial blood-brain barrier (BBB), the blood-leptomeningeal barrier (BLMB), and the blood-CSF-barrier (BCSFB) are considered most relevant. The BBB is formed by endothelial cells, astrocytes, and pericytes in and around microvessels that reach into the CNS parenchyma. The BLMB is established by a thin layer of cells of the pia mater that cover the surface of non-fenestrated microvessels in the subarachnoid space [14]. The BCSFB is located in the choroid plexus of the brain ventricles. It comprises choroid plexus epithelial cells which are connected *via* tight junctions and meningeal postcapillary venules that harbor a fenestrated endothelium [15]. In addition to the brain vascular system, recent research has identified a dural lymphatic system within the meninges, running along the dural sinuses and accounting for drainage of macromolecules and cells from the deep parenchyma of the CNS [16, 17]. Accordingly, a potential blood-dural lymphatics barrier (BDLB) could hypothetically play a role in CNS infiltration besides the BBB, BLMB, and BCSFB.

### Barriers and shortcuts: routes for leukemia cells to infiltrate the CNS

*In vivo* studies with patient derived xenograft (PDX)-ALL cells in mice conducted in the last years collectively found that the brain parenchyma is rarely infiltrated by ALL cells and that if this happens, it occurs mostly in the final stages of CNS leukemia [18–22]. These observations may be limited by the artificial nature of the model systems available. However, they confirm findings from an early human study, which found parenchymal involvement only in 17 out of 126 autopsy brain samples and only in those with late stage disease [8]. Histopathological data indicates that in the final stages of CNS involvement, leukemic cells may expand along perivascular spaces that reach into the brain parenchyma (Virchow-Robin spaces) and eventually breach the pia-glial membrane to invade the cerebral cortex (Fig. 1, Route 1) [3, 23]. A recent study followed the engraftment of a GFP-labeled Nalm-6 ALL cell line *via* intravital microscopy and found that, analogous to metastasis models of solid malignancies, ALL cells are trapped in the branches of microvessels early after injection. However, unlike disseminated carcinoma cells, leukemia cells fail to enter the brain parenchyma [24]. These findings support the view that parenchymal involvement of ALL *via* entry of leukemic cells into the CNS through the BBB is probably less important. It therefore appears more likely that leukemia cells enter the CNS *via* the BLMB or the BCSFB (Fig. 1, routes 2 and 3). Indeed, various *in vivo* studies in xenograft models of ALL were able to locate CNS infiltrating cells in the subarachnoid space of the leptomeninges in proximity to the dural venous sinuses [18, 20, 22]. This is in line with Price and Johnson’s autopsy study, which found arachnoid involvement in 70 out of 126 autopsy brain samples [8]. In the early stages of leukemic involvement, the distribution of ALL cells was limited to the superficial arachnoid and the subarachnoid space [8]. Interestingly, the *in vivo* study already mentioned also found that Nalm-6 cells xenografted into NSG mice circulated through and shortly persisted in the leptomeningeal vasculature but did not cross the BLMB. The choroid plexus was also found to be free of leukemic cells until late stages of the disease, which also contradicts ALL entry into the CNS *via* the BLMB and BCSFB [24]. When seeking for alternative entry routes besides the BLMB and the BCSFB, Yao et al. detected minor cavities directly traversing the bone marrow and the subarachnoid space. These cavities co-stained with laminin and α-smooth muscle actin (αSMA) and were hence hypothesized to correspond to bridging veins. In leukemia-bearing mice, these cavities were indeed filled with ALL cells. Laminin is enriched in the abluminal (external) surface of blood vessels [25]. Yao et al. therefore hypothesized that integrin-laminin-mediated mechanisms were necessary for interaction with these vessels. Hence, it is possible that instead of choosing the passage *via* the BBB, BLMB, and BSCFB, leukemia cells utilize this direct shortcut along the surface of bridging veins to invade the subarachnoid space (Fig. 1, route 4). However, it remains to be seen if this infiltration route shown in preclinical mouse models is relevant in CNS infiltration in human ALL. Dissemination of leukemia cells into the CNS was thus long considered to occur *via* the blood. Nevertheless, the recently discovered dural lymphatic system represents a further route of lymphocyte trafficking [26], and it may be possible that ALL cells hijack the CNS lymphatics to enter or leave the CNS (Fig. 1, route 5), which may represent a promising novel research direction with profound clinical implications. This hypothesis is of particular interest in the context of CNS relapse, as leukemia cells may reconquer the systemic circulation, which could be why isolated CNS relapse patients almost always have minimal residual disease in the bone marrow and also require systemic therapy [2, 3]. Of note, none of the abovementioned studies shows evidence for the exclusivity of a particular entry route. Hence, it is also possible that ALL cells may use a number of different routes to invade the CNS at the same time—the BBB, the BLMB, the BCSFB, emissary veins and brain lymphatics. Moreover, the entry routes into the CNS may vary in different ALL subtypes (B cell precursor *versus* T cell disease, or, different cytogenetic backgrounds). Figure 1 summarizes the potential entry routes of ALL cells and their spatial distribution in the CNS.

## Encountering a new environment: cellular features promoting infiltration and survival in the CNS niche

It has been a matter of debate if the ability to invade the CNS is a common feature of ALL cells, or if some cells have a particularly high propensity to cause CNS leukemia due to their molecular features. Here, we will discuss some of the main molecular characteristics of ALL cells that may promote their entry and survival in the CNS niche.

### Migration and adhesion

Several studies have investigated whether ALL cells invading the CNS show a high migratory potential, for example through upregulation of chemokine receptors and/or adhesion molecules. Among lymphocytes, T-cells are more prone to enter the CNS than other lymphocytes. T-cells constitute the majority of lymphocytes in the physiological CSF, whereas the number of B-cells and NK-cells is comparatively low [27]. T-cells in the CSF express high levels of the adhesion molecules P-selectin glycoprotein ligand 1 (PSGL-1) and lymphocyte function-associated antigen-1 (LFA-1), by which they adhere to P-selectin and intercellular adhesion molecule-1 (ICAM-1), respectively, in the choroid plexus and subarachnoid space vessels [28]. Moreover, T-cells in the CSF express chemokine receptors including CXCR3, CCR5, CCR6, and CCR7 and the corresponding CCR7 ligands CCL19 and CCL21 have also been detected in choroid plexus epithelium [28]. This may be applicable to T-ALL also and the interaction of T-ALL cells with the choroid plexus and subarachnoid vessels *via* adherence and chemokine signaling may represent an important mechanism of CNS pathology. Indeed, the CCR7-mediated binding of T-ALL cells to CCL19 and CCL21 in the choroid plexus epithelium was demonstrated as a probable axis of CNS-infiltration by T-ALL cells [29]. More recently, caspase recruitment domain-containing protein 11 (CARMA1), which regulates the migration of physiological T-cells, was shown to be linked to infiltration of T-ALL [30].

In BCP-ALL, the general ability of leukemic cells to cause CNS leukemia in preclinical models has been advocated in a study by Williams et al.*,* in which 23 of 29 patient samples injected into immunedeficient mice (79%) caused CNS leukemia in the leptomeninges irrespective of their cytogenetic background and corresponding patient CNS status [22]. They also found that chemokine receptor signaling does not drive CNS entry in BCP-ALL as they found no differential chemokine receptor expression between non-CNS-homing *versus* CNS-homing BCP-ALL cells, supporting that the mechanisms of CNS infiltration of T-ALL do not necessarily apply to BCP-ALL [22]. A more recent study applying high-throughput sequencing techniques found that the clonal architecture of BCP-ALL in the CNS and bone marrow are similar, contradicting the hypothesis that clones with a particular CNS-tropism exist [31]. However, another study identified the upregulation of trafficking/adhesion markers, including CXCR3 and PSGL-1, in BCP-ALL populations isolated from the CNS compared to bone marrow-derived cells [32]. Furthermore, α6-integrin signaling was shown to enhance the migration of BCP-ALL cells towards CSF samples *in vitro*, serving as the molecular basis for ALL migration along bridging veins [24]. Overall, the significance of adherence and chemokine signaling in entry mechanisms of BCP-ALL cells into the CNS is not fully understood and requires further investigation.

### Survival pathways in the CNS niche

When entering the leptomeninges, ALL cells encounter a hostile microenvironment with diminished oxygen and nutrient supply. In addition to adhesion and homing mechanisms, ALL cells were shown to specifically upregulate pathways that help them colonize the CNS niche. Cytokines such as interleukin (IL)15 and IL7 are involved in lymphocyte development and are highly abundantly present in the CNS [33]. Both pathways were shown to be associated with CNS infiltration. IL15 was found to be upregulated in patients initially diagnosed as CNS positive (presenting with ALL CNS infiltration), this being predictive of CNS relapse [34]. Biologically, IL15 was found to promote the growth of primary BCP-ALL cells, particularly in low growth factor conditions as can be found in the CSF [35]. However, another study found that cells isolated from the CNS did not show enhanced IL15 expression as compared to ALL cells in the bone marrow [32]. Therefore, the role of IL15 signaling in the CNS is not yet fully elucidated. It was proposed that IL15 expression in BCP-ALL cells is important for the interaction with NK-cells, which are virtually absent in the CNS, indicating a possible immune escape strategy for ALL cells in the CNS niche [18]. More recently, high expression of the IL7 receptor (IL7R) in BCP-ALL cells from bone marrow/peripheral blood at diagnosis were shown to be associated with a positive CNS status and a higher risk for CNS relapse in patients [36]. Accordingly, treatment with an antibody blocking IL7R markedly reduced leukemic infiltration in different compartments including the CNS and significantly increased survival of NSG mice injected with cells from BCP-ALL patients [36]. The IL7R pathway is known to cooperate with the pre-B cell receptor (preBCR) pathway and their interplay is mandatory for the survival of pre-B-cells [37]. Recently, evidence supporting the view that preBCR signaling is involved in CNS involvement of BCP-ALL has been accumulating. In BCP-ALL PDX cells xenografted into mice, high expression of the Zeta-chain-associated protein kinase 70 (ZAP-70) which acts directly downstream the preBCR was found to be associated with the ability to engraft in the CNS [38]. Moreover, knockdown of ZAP-70 in the BCP-ALL cell line 697 resulted in a significant decrease of CNS disease *in vivo.* High ZAP-70 expression in diagnostic BM samples of BCP-ALL patients was associated with a 7.5-fold increased risk for CNS disease [38]. Further data suggest that not only the pathway downstream of the preBCR is involved in CNS engraftment, but also the receptor complex itself. *E2A-PBX1* positive PDX cells retrieved from the CNS of xenografted mice showed a significant enrichment for the preBCR signaling pathway and the upregulation of the preBCR signaling units CD79a and CD79b [39]. Knockdown of CD79a in *E2A-PBX1* positive 697 cells resulted in a clear reduction of leukemic engraftment restricted to the CNS niche. Notably, a high expression of CD79a as determined in diagnostic BM samples of pediatric BCP-ALL patients was associated with CNS infiltration upon diagnosis. PreBCR signaling may also be involved in the mechanisms of relapse in BCP-ALL. Good et al. identified a high basal activation level of preBCR-signaling and an absent response to stimulation of the preBCR pathway as a feature of BCP-ALL that predicts a higher likelihood of relapse [40].

Studies have shown that one of the key regulators in CNS infiltration *via* IL15 and ZAP-70 is the mitogen-activated kinase (MAP-kinase) extracellular-signal regulated kinase (ERK) [35, 38]. The RAS-RAF-MEK-ERK pathway represents one of the major axis for preBCR-mediated signaling transduction [41]. In addition, a study by Gaynes et al. found the upregulation of RAS and MAPK pathway genes in ALL cells recovered from the CNS, as compared to bone marrow ALL cells [42]. Activating mutations in this pathway have already been shown to be associated with an adverse clinical phenotype including an enhanced frequency of CNS involvement [43, 44]. Therefore, active ERK signaling may be a key feature of ALL cells surviving in the CNS. Cellular adaptation to hypoxic conditions appears to be a further important survival mechanism for leukemic cells in the CNS. In two recent independent studies, ALL cells isolated from the CNS were shown to upregulate genes associated with hypoxia adaption [20, 45]. One of the key molecules identified in both studies was vascular endothelial growth factor (VEGF) [20, 45]. Blockade of VEGF *via* the approved anti-VEGF antibody bevacizumab resulted in diminished leukemic engraftment in the CNS, but not in other tissues in PDX models of ALL. VEGF was also shown to enhance migration through a microvascular endothelial cell layer *in vitro,* which also indicates that next to survival signaling, VEGF may also be important for ALL entry into the CNS [20].

### The relevance of cytogenetics

A T cell immunophenotype is generally considered a risk factor for CNS infiltration in ALL [2]. Only a few genetic abnormalities have been shown to promote CNS-infiltration of T-ALL cells. For example, activating mutations of the Notch signaling pathway are found in ~ 80% of T-ALL patients [46] and ectopic expression of Notch resulted in a T-ALL phenotype with potent infiltration of the leptomeninges in mouse models [29]. However, a systematic investigation of CNS tropism of T-ALL cells has not been conducted to date, and it is possible that CNS tropism may also be associated with particular T-ALL cytogenetic subtypes.

Compared to T-ALL, the molecular characterization of genetic aberrations is more advanced in BCP-ALL and has led to the identification of various gene fusions with diagnostic and clinical relevance [47]. This progress in the molecular characterization of BCP-ALL also holds the potential to deepen the understanding of the molecular background of CNS disease in ALL. The t(1;19) translocation leading to the *E2A-PBX1* fusion gene is found in 5–10% of BCP-ALL patients [48]. It was recently shown that the *E2A-PBX1* fusion can readily emerge *in utero* during fetal hematopoiesis but remains clinically unobtrusive. Secondary genetic alterations may then contribute to malignant transformation of *E2A-PBX1*-positive preleukemic clones [49]. It was shown that patients with *E2A-PBX1* fusion gene have increased frequencies of CNS positive patients upon initial diagnosis [19] and relapse [11]. Investigations conducted in the recent years have shed some light into the molecular backgrounds for the particular CNS tropism and relapse of *E2A-PBX1*-positive leukemia. The proto-oncogene tyrosine-protein kinase (TK) MER was among the first identified targets for CNS involvement with a particular significance in *E2A-PBX1*-positive leukemia [19]. MER-TK was found to be upregulated in 64 *E2A-PBX1*-positive BCP-ALL patients compared to 93 patients of other cytogenetics. Accordingly, down-regulation of MER led to a decrease of CNS involvement in xenografted immunodeficient mice. However, there is no evidence suggesting that MER is directly controlled by *E2A-PBX1* [10, 19]. It was also shown that *E2A-PBX1* causes alterations in IL7R signaling [50] and high expression of the Interleukin-7-receptor (IL7R) in *E2A-PBX1*-positive ALL compared to other cytogenetics had previously been shown in two independent series [51, 52]. Given the importance of IL7R in CNS leukemia, IL7R-mediated CNS infiltration could be of particular importance for *E2A-PBX1* positive leukemia. Furthermore, the transcription factor PBX1 was shown to be associated with survival of ALL cells in the CNS [42]. Hence, in *E2A-PBX1* positive leukemia, PBX1-dependent survival signaling may be particularly enhanced and promote CNS tropism.

A further genetic abnormality associated with an enhanced risk for CNS infiltration is the t(9;22)(q34;q11.2) translocation leading to the *BCR-ABL* fusion gene (Philadelphia chromosome positive BCP-ALL). The *BCR-ABL* fusion is detected in about 3–5% of pediatric BCP-ALL patients [1, 53]. Patients with *BCR-ABL*-positive leukemia represent a subgroup with a particularly poor outcome due to a higher incidence of relapse and refractory disease [54]. CNS involvement is detected in about 6% of patients rendering *BCR-ABL*-positive ALL a CNS high risk cytogenetic subtype [55]. The application of specific tyrosine kinase inhibitors like imatinib revolutionized the therapy of *BCR-ABL*-positive leukemia; however, 15–20% of patients show relapses under therapy, including in the CNS [56]. This is to some extent due to the poor ability of imatinib to penetrate the BBB [54]. Yet, the *BCR-ABL* fusion gene may provide cells with a particular fitness by helping them to persist and survive in the CNS niche under therapy. The molecular backgrounds of CNS disease in *BCR-ABL*-positive ALL remain poorly characterized. However, it appears that CNS disease in *BCR-ABL*1-positive ALL shares some common features and pathways with other cytogenetic subtypes with CNS tropism. Different *in vivo* model systems of *BCR-ABL*-positive ALL based on the introduction of a *BCR-ABL*1 fusion gene into murine bone marrow cells showed profound infiltration of the CNS [39, 57]. One study in a mouse model of *BCR-ABL* positive leukemia with CNS tropism found the upregulation of L-selectin and integrin subunit alpha 6 (Itga6) in *BCR-ABL* expressing cells compared to control cells [57]. By this, *BCR-ABL*-positive cells may be prone to adhere to CNS vessels and to utilize the vascular entry routes described above. It was moreover shown that Wnt/Ca2+/NFAT signaling promotes the survival of *BCR-ABL*-positive chronic myeloid leukemia cells upon inhibition of *BCR-ABL1* and that NFAT inhibition enhanced the susceptibility of *BCR-ABL* positive ALL cells to imatinib [58]. Wnt and NFAT signaling have been strongly implicated in the development and homeostasis of the CNS [59]. Hence, one can speculate that the *BCR-ABL* fusion may render leukemia cells receptive to survival signals, which could also be derived from the CNS niche.

About 10% of childhood leukemias have a gene expression profile similar to *BCR-ABL*-positive leukemia although lacking the actual BCR-ABL fusion protein. These ALLs are therefore termed the *BCR-ABL*-like class [60]. The increasing molecular understanding of *BCR-ABL*-like ALL identified rare cytogenetic subtypes in this group, which may also be associated with a particular risk of CNS infiltration [47, 60]. Transcripts involving the *NTRK3* gene are involved in the malignant progression of different solid cancers [61]. Moreover, *NTRK* fusions were shown to be present in ~ 1% of *BCR-ABL*-like BCP-ALLs [62, 63]. An *in vivo* system modeling *ETV6-NTRK3* positive leukemia exposed a particularly aggressive clinical phenotype with excessive CNS infiltration [62, 63]. In a recent report of pediatric ALL, it was shown that the TRK-inhibitor larotrectinib may cause long-lasting remissions in *ETV6-NTRK3* positive BCP-ALL with multiple CNS relapses [64]. The molecular background of *ETV6-NTRK3*-positive BCP-ALL and its CNS tropism yet remain elusive. Roberts et al. showed in their mouse model that *ETV6-NTRK3*-positive cells exposed upregulation of genes of the activator protein (AP-)1 pathway [62], which may represent one reason for the CNS infiltrating capacity of *NTRK3* fusion positive ALL.

Rearrangements of *MLL* (*MLL*r) (also known as *KMT2A*) represent a poor prognosis cytogenetic subtype of BCP-ALL. *MLL*r*-*ALL shows a pro-B/mixed phenotype and frequent therapy resistance [47, 65]. The CNS is involved in about 5% of *MLL*r BCP-ALL patients [66]. *MLL*r-ALL have frequent mutations in the RAS pathway [67–69], which as previously discussed was shown to be linked with an increased risk of CNS infiltration [44]. Furthermore, KRAS activation was shown to promote CNS infiltration of *MLL*r cells harboring the *MLL–AF4* fusion gene [70]. Accordingly, the MEK inhibitor trametinib impacted ERK phosphorylation and decreased CNS infiltration of the *MLL*r KOPN8 ALL cell line transplanted into NSG mice [71]*.* Thus, Ras mutations might in part explain the high rates of CNS infiltration in this particular disease subtype, and targeting this pathway may be a therapeutic option worth exploring. It was recently shown that a high expression of neuron-glial antigen 2 (NG2) was associated with decreased event-free survival and with a high WBC in a small patient cohort of *MLL*r patients. In an *in vivo* mouse model*,* only NG2-positive cells were found in the CNS even if NG2 low cells were initially transplanted, indicating a neurotropic function of NG2 in *MLL*r BCP-ALL [21]. However, blockade of NG2 by different means had no effect on CNS infiltration and evidence for NG2 being relevant for CNS involvement in patients is limited. Both, KRAS activation and NG2 upregulation were found to be associated with a migratory gene signature [21, 70]. Hence, further studies are necessary to validate if migration/adhesion processes play a direct role in the ability of *MLL*r ALL cells to invade the CNS.

Taken together, the different cytogenetic ALL subtypes at high risk for CNS involvement show an upregulation of pathways promoting entry and survival in the CNS niche. Some pathways for CNS tropism may thereby be of particular or even exclusive relevance in specific cytogenetic subtypes. The ongoing characterization of biological peculiarities of high risk cytogenetics may help to identify further pathways involved in CNS tropism and also identify targets with a therapeutic relevance.

**Fig. 2:**
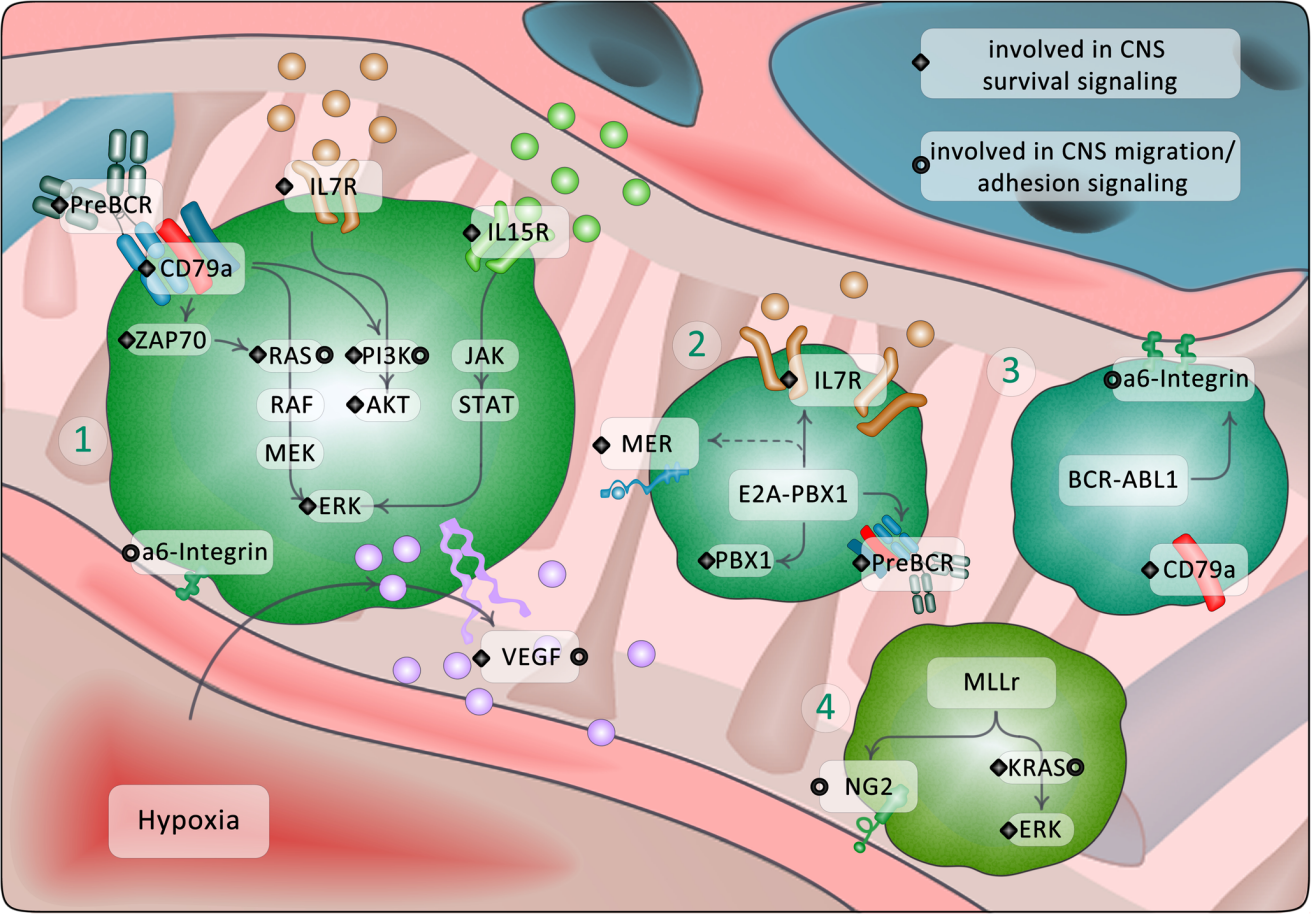
Pathways and molecules in CNS infiltration and survival. When reaching the CNS, ALL cells have to cope with a hostile microenvironment. Upregulation of migration/adhesion and survival pathways may cause a particular CNS tropism in ALL cells. Some pathways (1) may be of general relevance for CNS involvement in BCP-ALL. Others may be of particular importance in ALL subtypes with a higher risk for CNS involvement due to their cytogenetic background like *E2A-PBX1* (2)*, BCR-ABL* (3), or *mixed lineage leukemia-*rearrangements (*MLL*r*)* (4). Molecules shown to be directly involved in CNS leukemia are marked with a circle (adhesion/migration signaling) or square (survival signaling)

Molecules and pathways hypothesized or shown to be associated with CNS involvement in BCP-ALL are depicted in Fig. 2.

## Silent sanctuary: dormancy as a mechanism of tumor survival and relapse in the CNS

We discussed potential ways of leukemia cells to enter the CNS, which pathways are involved in CNS leukemia, and how cytogenetics may contribute to an enhanced potential for CNS infiltration. In the following section, we would like to speculate on the biological behavior of leukemia cells in the CNS, stimuli that may cause or prevent relapse, and how the cellular behavior is shaped by the microenvironment with particular regard to cellular dormancy.

Leukemia cells invading the CNS encounter conditions that differ from the milieu a leukemic cell is exposed to when circulating through the vasculature or when residing in the bone marrow. For disseminated carcinoma cells, three different fates have been hypothesized [72], which may also apply to leukemia cells in the CNS: first, and most likely for many cancer cells, a leukemic cell is unable to rapidly adapt to the CNS microenvironment and goes into apoptosis. Second, a leukemia cell or a fraction of leukemia cells might be able to enter a state of quiescence, in which they are able to reside in the secondary microenvironment for longer periods of time without being adversely affected by systemic therapy. The third option is that a clone with particular fitness or a quiescent cell receiving pro-proliferative stimuli from the microenvironment starts to proliferate in the CNS and forms a clinically relevant lesion [72], in this case overt CNS leukemia. Although these hypotheses were initially generated for disseminated cells of solid malignancies, which might have to undergo further adaptive processes (e.g., mesenchymal-to-epithelial transition, MET [72]), it is likely that corresponding rules apply to cancer cells derived from hematological malignancies in unfavorable niches. Cellular dormancy describes cells resting in a reversible G0/G1 arrest of the cell cycle with diminished metabolic activity [73]. Dormant solid tumor cells are furthermore characterized by a low expression of proliferation markers like Ki67 and a low ratio of phosphorylated (p-)ERK to p-p38 [74]. P38 was also shown to be an important survival pathway in ALL [75]. Indeed, it has been shown that ALL cells in the CNS may acquire a slow cycling phenotype. A study by Jonart et al. detected increased G0/G1 cell cycle arrest and decreased Ki67 positivity in ALL cells recovered from the meninges compared to cells in the peripheral blood and bone marrow [76]. Similar phenotypes were obtained when culturing ALL cells in the presence of primary meningeal cells *in vitro.* The anti-proliferative effect was less pronounced when incubating ALL cells with medium conditioned by meningeal cells, indicating that the quiescent phenotype of ALL cells is promoted by direct interactions with CNS cells rather than by secreted factors [76]. These results are in line with previous reports which found that ALL cells of different cytogenetic backgrounds go into G0/G1 arrest when brought in direct contact with cell lines representative of the CNS niche [19]. In the study by Jonart et al., the effects of CNS niche cells promoting quiescence were partially dependent on VCAM-1, which was downregulated in ALL cells when removing them from coculture conditions [76]. VCAM-1 and its interaction with integrin-α4β1-positive cells were shown to play a significant role in dormancy regulation in breast cancer models [77]. Furthermore, the interaction between VCAM-1 and tumor cells was shown to promote the upregulation of AKT [78]. Accordingly, the acquisition of a dormant phenotype was shown to depend on MER-TK and AKT signaling in *E2A-PBX1*-positive cells. The AKT pathway represents an integral survival pathways of tumor cells and was shown to play a role in CNS infiltration [19, 38, 44]. Therefore, one may speculate that adhesion processes of leukemic cells to particular cell entities in the CNS induce dormancy and concomitant survival signaling *via* AKT as an integral signaling component.

A further interesting mechanism in the context of CNS-mediated ALL dormancy could be the adaption to hypoxic conditions. In line with the previously mentioned report, Kato et al. showed that ALL cells from a CNS relapse patient injected into mice and recovered from the CNS expose a lower Ki67 index and proportion of cells in the S/G2/M phases of the cell cycle than cells isolated from the bone marrow [45]. These cells moreover displayed diminished oxygen consumption indicative of lowered mitochondrial activity. Comparative transcriptomic analyses between PDX cells from the CNS and the bone marrow found that besides the downregulation of cell cycle genes, CNS-ALL cells expressed a hypoxia-associated gene signature [45]. Accordingly, in solid malignancies, hypoxic conditions were shown to foster a dormant phenotype and the upregulation of key dormancy genes like *DEC2*, *p27*, and the hypoxia genes *GLUT1* and *HIF1* [79]. Postulating that the mechanisms of hypoxia-mediated dormancy are similar in solid malignancies and in ALL, this may represent an interesting approach for new targeted clinical approaches. Therapy resistance and relapse are the main causes of therapy failure in ALL [1]. It is commonly acknowledged that the CNS represents a sanctuary with limited immune surveillance, in which ALL cells may reside protected from therapeutic damage. However, the clinical observation of isolated CNS relapses even years after complete remission indicates that ALL cells may persist in the CNS over long periods of time [11, 80]. Furthermore, quiescent and chemoresistant leukemia cells have been identified as a potential source of minimal residual disease (MRD) after therapy [81]. Independent reports found that direct contact with CNS cells promoted resistance of ALL cells to chemotherapeutic drugs including methotrexate [19, 42, 76], cytarabine [42, 76] and dexamethasone [82]. Chemoresistance may be partially promoted by the transcription factor PBX1, which was found upregulated in leukemia cells in the CNS compared to bone marrow ALL cells [42]. In most mentioned studies, the chemoresistant phenotype was lost when recovering cells from coculture conditions. An elegant study based on tracing labeled PDX-ALL cells *in vivo* identified rare long-term dormant and therapy refractory cell subpopulation that resembles dormant leukemia cells [81, 83]. These cells reacquired a proliferative phenotype and lost their drug resistant profile when detached from their environment [83]. These observations may also be applicable to ALL cell populations the CNS and indicate that a particular dormant subset of leukemia cells residing in close proximity to CNS cells may represent a refractory fraction and the origin of relapse in the CNS.

Overall, it appears that dormancy represents an important mechanism of persistence and recurrence of ALL in the CNS. Mobilizing and thereby activating leukemia cells could enhance their susceptibility to chemotherapy [84]. However, more research is needed to determine if processes associated with dormancy could represent clinically relevant therapeutic targets.

## New perspectives in the treatment of CNS leukemia

We have discussed the recent progress in the molecular background of CNS infiltration and relapse in BCP-ALL. We will now review different emerging (immune) therapy options beyond conventional chemotherapy that have already become available for other indications or may hold the potential to enter clinical application in ALL with an involvement of the CNS.

One option to prevent and eradicate CNS leukemia is the use of inhibitory substances that target pathways associated with CNS entry of ALL cells or their survival in the CNS niche. An interesting example in this regard could be the PI3K inhibitor idelalisib. PI3K was shown to be involved in integrin-mediated adherence to CNS vessels and its blockade with idelalisib resulted in clear and specific reduction of CNS involvement in a preclinical model [24]. Furthermore, PI3K is a key molecule of the preBCR signaling machinery, which was shown to represent a potential target in BCP-ALL [85], and for which an association with CNS involvement was suggested [39]. PreBCR-dependent BCP-ALL cells showed a particular sensitivity to idelalisib treatment [86, 87]. Of note, combination therapies including idelalisib are already being tested in clinical studies of other hematological malignancies such as chronic lymphocytic leukemia [88]. PI3K is also critically involved in IL7 signaling, which is vital for B cell differentiation and survival [89].

In preclinical models, a further substance, the JAK inhibitor ruxolitinib was inferior in targeting BCP-ALL cells in the CNS compared to a monoclonal antibody against IL7R [36], but first case reports show that ruxolitinib can be clinically efficient in BCP-ALL. The combination of ruxolitinib with chemotherapy resulted in molecular remission in a high risk *BCR-ABL*-like refractory patient with macroscopic CSF involvement [90]. A further case report showed eradication of refractory ALL in the CNS [91]. Ruxolitinib is currently being tested in a larger clinical study (AALL1521), which will yield a clearer understanding of the impact of this drug on ALL including CNS involvement.

A further approach that may improve the treatment of CNS leukemia is the use of antibody-based immunotherapy, which is evolving quickly. The bispecific T cell engager (BiTE) blinatumomab has gained FDA approval and is increasingly being applied in relapsed and refractory ALL in adults and children [92]. However, the evidence for the efficacy of blinatumomab in CNS leukemia is poor, as patients with overt CNS pathology were excluded from clinical studies. Nevertheless, single case reports indicate that blinatumomab may represent a tolerable therapy option for patients with a history of CNS disease when applied with concomitant CNS-targeted chemotherapy [64, 93]. The ability of blinatumomab to penetrate into the CNS is discussed controversially. One the one hand, displaying a molecular weight of 55 kDA [94], blinatumomab is a particularly small construct and may be able to traverse brain barriers on its own even though antibodies are usually not able to shuttle to the CNS. Furthermore, it is hypothesized that blinatumomab activates T cells which then adhere to the endothelium, breach the BBB, and exert cytotoxic effects on CD19 positive and BBB cells [92]. The efficacy of antibody-based immunotherapies in relapsed and refractory BCP-ALL is evident, but knowledge on their effect on CNS disease is limited. Limited efficacy in the CNS is probably due to their poor penetration into the CNS which can, however, be overcome by intrathecal or intraventricular application [95, 96]. Indeed, treatment of pediatric CD20-positive B cell lymphoid malignancies including relapsed ALL *via* intrathecal injection of rituximab resulted in complete CNS remission in the majority of patients, with an acceptable toxicity profile in small-sized clinical studies [97, 98]. Further approaches for antibody-based immunotherapy are on the way. Promising preclinical reports showed the efficacy of the monoclonal CD19 antibody CD19-DE in BCP-ALL [99] and the CD38 antibody daratumumab in T-ALL [100, 101] and first clinical trials have been initiated (e.g., NCT03384654). Other antibodies are increasingly being used in clinical routine like the CD20 antibody rituximab and the CD22 antibody inotuzumab-ozogamycine. Novel surface targets accessible to therapeutic antibodies (e.g., the IL7R [36] and VEGF [20, 45]), are emerging. In order to overcome the poor ability of antibodies to reach the CNS, engineering approaches to promote BBB crossing may come into focus of research. Equipping antibodies with domains that promote CNS shuttling mechanisms may increase their potency to reach cells residing in the CNS. Such approaches have shown promising results in preclinical models of brain metastases of solid tumors [102]. It will be interesting to see if antibody-based immunotherapy will enter clinical practice in the treatment of CNS leukemia.

A potential additional way to cross anatomical barriers in the brain is the application of cellular therapies. To this end, a promising approach appears to be the application of chimeric antigen receptor (CAR)-T cells. CAR-T cells are generated by genetic engineering of a patient’s own T cells. CARs consist of elements of the T cell receptor complex like the CD3ζ chain and the costimulatory domains CD28 or 4-1BB and a tumor-antigen-specific monoclonal antibody domain. The most common target for approved CAR-T-cell products in the treatment of hematological malignancies is CD19 (e.g., tisagenlecleucel). Their application spectrum includes diffuse large B cell lymphoma (DLBCL) and relapsed/refractory BCP-ALL. CAR-T-cells have shown impressive response rates in adult and pediatric patients [103, 104]. Due to concerns regarding neurotoxicity, initial clinical studies with CD19 CAR-T cells strictly excluded patients with CNS involvement [105]. However, more recent evidence from first clinical studies or off-label use, suggest that CAR-T-cells may represent an efficient option for treating CNS leukemia. Independent reports including small patient numbers showed sustained complete remission after CD19 CAR-T cell treatment in ALL patients with refractory disease and CNS involvement and/or isolated CNS disease [103, 106, 107]. Furthermore, the neurotoxic profile of CAR-T cells in patients with CNS involvement was limited and reversible [103, 106, 107] and, most importantly, did not differ from patients without CNS disease. However, systematic studies with larger patient numbers are needed to assess the general benefit of CAR-T-cells in CNS leukemia. Corresponding studies with CD20-CAR-T cells could follow and may be of particular interest in BCP-ALL and other hematological malignancies in the brain, such as primary CNS lymphoma. As CD19 is apparently variably expressed in the CNS, including neuronal structures [108], CD20 appears to be a more suitable target in treating CNS disease due to a potentially lower neurotoxicity profile. It can be assumed that CAR-T cell therapy to treat CNS involvement will become increasingly common, warranting further clinical studies and diminishing therapy costs.

Altogether, new and promising approaches to cure CNS involvement are on the way. Modified immune therapies or niche targeting strategies may enhance the efficacy of existing therapy protocols or even replace them in the future. There will be an increased need for preclinical evaluations and clinical studies for novel therapeutic agents in patients with leukemic involvement of the CNS.

## Conclusion

CNS disease is an unresolved problem in the treatment of ALL due to the fact that current CNS directed therapy is unspecific and can be toxic. In order to develop novel diagnostic and therapeutic strategies, it will be increasingly important to understand the molecular mechanisms of CNS infiltration in ALL. This must include research on entry routes, survival pathways, and niche-dependent cellular behavior in the CNS. For that, further development of adequate preclinical models is essential. Novel targeted therapies including immunotherapies will become increasingly important in the treatment of ALL, including CNS leukemia, and potentially enhance the efficacy of existing therapies. Furthermore, targeted therapies with lower toxicity may spare patients acute and long-term therapy toxicities in the future.
